# HotSpotAnnotations—a database for hotspot mutations and annotations in cancer

**DOI:** 10.1093/database/baaa025

**Published:** 2020-05-08

**Authors:** Victor Trevino

**Affiliations:** Tecnologico de Monterrey, Escuela de Medicina, Cátedra de Bioinformática, Morones Prieto No. 3000, Colonia Los Doctores, Monterrey, Nuevo León 64710, Mexico

## Abstract

Hotspots, recurrently mutated DNA positions in cancer, are thought to be oncogenic drivers because random chance is unlikely and the knowledge of clear examples of oncogenic hotspots in genes like BRAF, IDH1, KRAS and NRAS among many other genes. Hotspots are attractive because provide opportunities for biomedical research and novel treatments. Nevertheless, recent evidence, such as DNA hairpins for APOBEC3A, suggests that a considerable fraction of hotspots seem to be passengers rather than drivers. To document hotspots, the database HotSpotsAnnotations is proposed. For this, a statistical model was implemented to detect putative hotspots, which was applied to TCGA cancer datasets covering 33 cancer types, 10 182 patients and 3 175 929 mutations. Then, genes and hotspots were annotated by two published methods (APOBEC3A hairpins and dN/dS ratio) that may inform and warn researchers about possible false functional hotspots. Moreover, manual annotation from users can be added and shared. From the 23 198 detected as possible hotspots, 4435 were selected after false discovery rate correction and minimum mutation count. From these, 305 were annotated as likely for APOBEC3A whereas 442 were annotated as unlikely. To date, this is the first database dedicated to annotating hotspots for possible false functional hotspots.

## Introduction

Cancer is a genetic disease in which mutations accumulate (1). Nevertheless, not all mutations are oncogenic because many mutations can be the result of broken DNA repair systems or expositions to mutagens (2,3). Therefore, it is fundamental to distinguish between oncogenic *driver* mutations and random *passenger* mutations. Instead of detecting specific mutations, many methods are focused on the gene level to identify putative driver genes (4–11). The most widely used method concentrates on those genes whose mutation frequency across patients is higher than random chance, correcting for gene length, background mutation rate and other covariates (4). Other methods center on the successful evolutionary concept that ratios of non-synonymous mutations over synonymous mutations (dN/dS) different to 1 identify those genes under positive or negative selection (12). Driver genes are highly enriched in those whose dN/dS > 1 (12).

Observing multiple patients mutated at the same nucleotide or amino acid position is known as *hotspot*. Under some statistical models, hotspots are highly unlikely and therefore commonly and irrefutable thought as drivers (13). This is also supported by several examples. For instance, *BRAF* at valine position 600 (p.V600) is the most recurrent known hotspot that has been validated to be oncogenic (14). In the database used here, there are more than 500 patients across diverse cancers reporting the *BRAF* V600 mutation. Similarly, *IDH1* at the arginine 132 (p.R132) is also a well-known oncogenic driver (15,16). Computational analyses and filtering of missense and nonsense mutations have identified more than 470 putative hotspots across hundreds of coding genes (13). Moreover, further recurrent positions are expected because tumor sequencing is expected to become an important tool for research and treatment (17).

Hotspots are very attractive for biomedical research because they provide opportunities to understand their oncogenic mechanisms and highlight possible targets or strategies for treatment and prevention (18). Some methods have been proposed to detect hotspots (13,19–22). The overall rationale is that hotspots should show a number of occurrences that is highly unlikely, commonly estimated under a certain model.

Nevertheless, it is important to note that detecting a mutation hotspot is not necessarily equivalent to detect a driver mutation. Seminal work has shown that recurrent mutations surrounded by a strong hairpin structure may be caused by the enzymatic activity of APOBECA (13). The authors showed that well-known cancer hotspots do not contain hairpin structures and that many apparent hotspots possessing hairpins appear in many not cancer genes (13). These results provide important mechanistic evidence that some hotspots did not arise by a functional pressure and therefore must be qualified as *passenger* hotspots. Consequently, to direct research into the more likely genuine targets, it is essential to distinguish between *driver* and *passenger* hotspots. Thus, models and databases for hotspots are highly valuable.

Although a database for hotspots is available (13), it does not keep track of their level of ‘*drivenness’* or ‘*passengerness’*. Besides, the method involved a filtering criterion either computational or manual that may remove hotspots without mechanistic evidence. Moreover, they used a binomial model corrected by some covariates. The use of the binomial may falsely call genes because the presence of over-dispersion cannot be handled (23), while the use of non-mechanistic covariates, although it helps the fitting, does not provide evidence of the mechanistic phenomena that give rise the hotspot.

To fulfill the lack of a database that annotates possible false functional hotpots, the hotspot database *HotSpotAnnotations* was created. To detect hotspots robustly, a mixed model of beta-binomial with a fixed effect was used (24). Then, up to now, detected hotspots have been or can be annotated by three methods that warn users as a possible passenger hotspot: first, by using the APOBEC3A method looking for *stemness strength* at strong hairpins and rules depending on loop length and position (18); second, by using the gene-level estimation of *dN/dS* showing that the gene has a different ratio of non-synonymous mutations suggesting non-neutral selection (12); and third, by allowing the community to add annotations to specific hotspots. Also, for future updates, it is planned to implement novel methods that explain mechanical effects when published. Accordingly, it is hoped that researchers will focus on hotspots that do not show evidence of possible mechanisms that generate functionally false hotspots.

## Methods

### Cancer data

Mutations were obtained from the public cancer repository TCGA (http://firebrowse.org/) on January 2018 corresponding to 33 cancer types, 10 182 patients, and 3 175 929 mutations (Table 1).

**Table 1:** Cancer data used to estimate hotspots

**Cancer**	**Patients**	**Mutations**	**Cancer**	**Patients**	**Mutations**	**Cancer**	**Patients**	**Mutations**
ACC	92	10 747	KIRC	336	26 693	PRAD	495	29 286
BLCA	412	134 513	KIRP	281	23 765	READ	137	64 804
BRCA	986	120 988	LAML	143	9905	SARC	237	28 159
CESC	289	103 405	LGG	508	35 556	SKCM	467	392 571
CHOL	51	5503	LIHC	364	54 238	STAD	437	213 144
COAD	399	264 786	LUAD	567	208 180	TGCT	144	3198
DLBC	37	6406	LUSC	492	181 116	THCA	492	10 899
ESCA	184	45 313	MESO	82	3827	THYM	123	4737
GBM	393	82 765	OV	436	75 168	UCEC	530	886 377
HNSC	508	102 309	PAAD	178	29 959	UCS	57	10 449
KICH	66	2896	PCPG	179	2411	UVM	80	1856

### Estimation of hotpots

The details of the method are available elsewhere (24). Briefly, a beta-binomial model has been used to estimate hotspots. The beta-binomial can capture over-dispersion commonly present in binomial data (23) and has been used to estimate recurrent alterations (25–27). The protein position as reported in raw data was used to aggregate mutations at the same position. The transcript ID that provides the protein position is shown in the user interface. Only mutations providing a protein position were used. A histogram having *k* bins was determined by counting the number of mutations observed per position. Then, an algorithm was applied that uses a mixed model with fixed effects. In this way, *h_i_ = BetaBin(a,b)_i_ + F_i_* where *h_i_* is the number of sites observing *i* mutations, *i = 0..k*, and *F_i_* is the fixed effect. The presence of hotspot mutations should increase the counts at some *i*. Thus, the algorithm first fit a beta-binomial model to the observed counts using a classical numerical method (‘L-BFGS-B’ for function *optim* in *stats* package in R, https://cran.r-project.org/). Then, the fixed effects are estimated by a stepwise algorithm. In each step, a matrix is built representing fractions of each count in columns, from 0.1 to 1 by 0.1 increments, and the *k* histogram counts in rows. At each matrix cell, the histogram is decreased by the corresponding *i* mutations and fraction of observed mutations to estimate its corresponding beta-binomial model. The best or *fittest* coordinate is used and aggregated to a fixed effect *F_i_* for the *i* mutation position. The stepwise algorithm stops when no or little improvement is observed using a Kullback–Leibler (KL) divergence measure, which occurs when KL is lower than 1, or when the number of steps is larger than three times the number of histogram bins. The fixed effects *F* absorbs those positions that cannot be explained by the beta-binomial model alone. Thus, the fixed effect vector *F* mark hotspots while the fitted beta-binomial estimate its probability, which were further corrected by a false discovery rate (FDR) approach (28) if their recurrence were at least 4.

### Annotation of APOBEC3A stemness

Following the original algorithm from authors (18), stem strength (*ss*) was estimated first calculating all combinations of loop lengths from 3 to 11 and mutation loop position from 1 to loop length, then obtaining the maximum stem strength along with its corresponding loop length and loop position. In case of ties, the minimum loop length and loop position was preferred. The *ss* is a score of the sum of nucleotide matches weighting 3 for GC pairs and 1 for AT pairs. So, *ss = 3xGC + 1xAT*. As in Buisson *et al.* (18), if the original mutation position refers to a G or T, the reverse complement of the sequence was used. To determine which *ss* values are likely to be APOBEC3A artifacts, Buisson *et al.* (18) stratified *ss* along with values of loop length, mutation position within the loop and sequence context. Thus, to facilitate the interpretation, an ‘APOBEC3A Hairpin’ value was added to hotspots. For this, the ‘Likely’ value was assigned to those *ss* values surpassing a threshold representing an excess of mutations given by APOBEC3A as shown in Supplementary Figure 9a from Buisson *et al.* (18) independently of the sequence context (because it is only defined for TC and only 1 of 16 combinations is not likely harpin with apparent low coverage compared to other combinations). The thresholds for *ss* used were 7, 12, 11, 12, 15, 14, 18, 15 and 16 corresponding to the combinations of *loop position*:*loop length* of 3:3, 3:4, 4:4, 4:5, 4:6, 5:6, 4:7, 5:7 and 6:8. For example, if the loop length is 3 and loop position was 4, the stem strength has to be higher or equal to 12 to be ‘Likely’ an APOBEC3A hairpin. Similarly, an ‘Unlikely’ value was assigned to those *ss* whose loop length and mutation loop position are amenable to APOBEC3A but whose *ss* do not cross a minimum threshold (threshold marked a ‘red’ in Supplementary Figure 9a from Buisson *et al.* (18)). Otherwise, a ‘No’ value is assigned to those that are not amenable to be a target of APOBEC3A. Besides the above annotations, the estimated values of *stem strength (ss), loop position* and *loop length* were added to the web interface after clicking the ‘+’ sign.

### Annotation of the evolutionary measure dN/dS

To estimate positive, negative or neutral evolution, the recent ‘CV’ method included in the package *dNdScv* was used (12). For this, mutations whose consequence were missense, nonsense, non-stop, silent, splice, frame shifts or start sites were considered. The *dndscv* function reports four estimations of *dN/dS* depending on the substitution type corresponding to missense, non-sense, splicing, and indels respectively (*wmis, wnon, wspl* and *wind*). All these fields and their corresponding *P* values can be viewed after clicking the ‘+’ sign that shows these and other details (see Results). As a quick reference in the main table, the maximum values of *wmis* or *wnon* were used as an overall dN/dS estimation.

**Figure 1:**
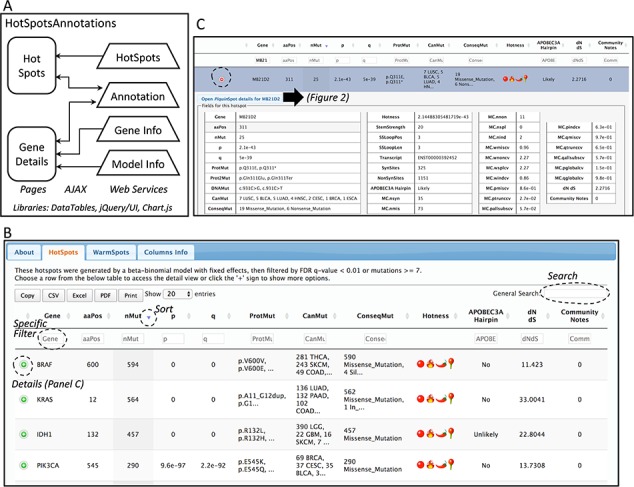
Database web implementation. (**A**) Implementation. There are two web pages, ‘Hot-Spots’ which list all hotspots, and ‘Gene Details’ which shows the details for the gene including all mutations and hotspots. There are four web services that provides the data for the pages (HotSpots, Annotation, Gene Info and Model Info). Examples of the web pages are shown in **B** and **C** and in Figure 2. (B) The first web page listing all hotspots. The list can be filtered, searched, exported and selected to open details. (C) Example of the estimation details for a hotspot.

### Database web implementation

The web site to access the generated database consists of two web pages. Both web pages use the libraries from *DataTables* (http://datatables.net), jQuery (http://jquery.com) and JQuery user interface (http://jqueryui.com). The first page includes all detected hotspots. The second page shows details per gene including the information of the fitted beta-binomial model, which makes use of Chart.js (https://www.chartjs.org/) for a graphical representation of the model. These pages are implemented in Java Server Pages under the GlassFish (https://javaee.github.io/glassfish/) application server. To facilitate loading, information is read from text-based databases. For community annotation of hotspots, gene and model information, web services provide the data needed to AJAX requests implemented in both pages. A schema of the implementation is shown in Figure 1A.

## Results

The overall process performed in this study is (i) hotspot detection, (ii) hotspot annotation for possible hairpins and dN/dS and (iii) web interface implementation.

### Hotspot detection

Putative hotspots were detected by the fixed effect, then annotated for the *P* value designated by the fitted beta-binomial distribution. The 23 198 ‘*warm’* spots having four or more mutations were further corrected by FDR and filtered by *q < 0.01* or observed in seven or more patients. Thus, the total reported possible hotspots were 4435 comprising 3384 genes. Both sets, hotspots and ‘warm’ spots, can be revised in the web pages. Although the concepts of ‘hotspot’ and ‘driver gene’ are not equivalent, they are related (see the Introduction section). Thus, to assess whether the 3384 genes carrying putative hotspots are enriched of driver genes, two comparisons with well-known cancer gene lists were made. The first comparison was made with the 221 reported in Martincorena *et al.* as under strong positive selection (12). The second comparison was performed with the 723 genes reported in the Cancer Gene Census form COSMIC (29). Both assessments were highly significant (*P* = 2e-62, *P* = 2e-50, hypergeometric test) suggesting that the hotspot database is highly enriched in cancer genes and cancer genes under positive selection.

**Figure 2:**
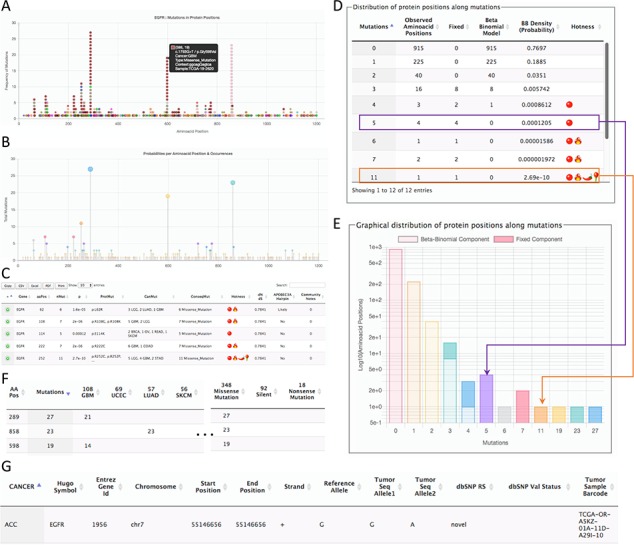
Page for the gene details. The example shows parts of the data shown for the gene EGFR. (**A**) All mutations in coding regions piled up per position. Pointing to a mutation, a floating tip show details of the mutation. (**B**) Raw count of mutations per position. (**C**) Detected hotspots for the current gene. (**D**) Distribution of mutations used to fit the beta-binomial model. (**E**) Graphical representation of the distribution of mutations and fitted model. (**F**) Mutations along cancer types per hotspot. (**G**) List of all mutations per gene.

### Annotation of hotspots

From the 4435 spots detected, 747 could be potential hairpins defined by the position of the mutation within the loop and the length of the loop following the parameters of Buisson *et al.* From the 747 spots, 305 indeed had a critical value of stem length forming a ‘*Likely’* hotspot as defined by Buisson *et al.* Thus, 442 spots were annotated as ‘*Unlikely’* and 3688 as ‘*No’* forming an APOBEC3A passenger spot. As a validation, some top genes mentioned by Buisson *et al.* were verified. Of the potential hotspots that were unaffected by the hairpin artifact include PIK3CA (N1044, 10 mutations), PIK3CA (E365, 10 mutations) and IDH1 (R132). These hotspots were also marked as ‘*Unlikely’* in our implementation because the stem strength was not strong enough to form a stable hairpin following the Buisson *et al.* criteria. Contrary, MB21D2 (Q311E, 25 mutations), FAM83G (Q88, 9 mutations), NUP93 (Q15, 7 mutations), MROH2B (E1109, 9 mutations) and C3orf70 (S6, 20 mutations) that were marked as optimal for the hairpin artifact in Buisson *et al.,* were also set as ‘*Likely’* by our implementation because stem strengths were above their corresponding thresholds. These observations suggest that our estimations are very similar to those of Buisson *et al.* An important gene to note by Buisson *et al.* was TBC1D12, which was not found in our database because only coding mutations were considered here while the hotspot in TBC1D12 is located at the 5′UTR.

**Figure 3:**
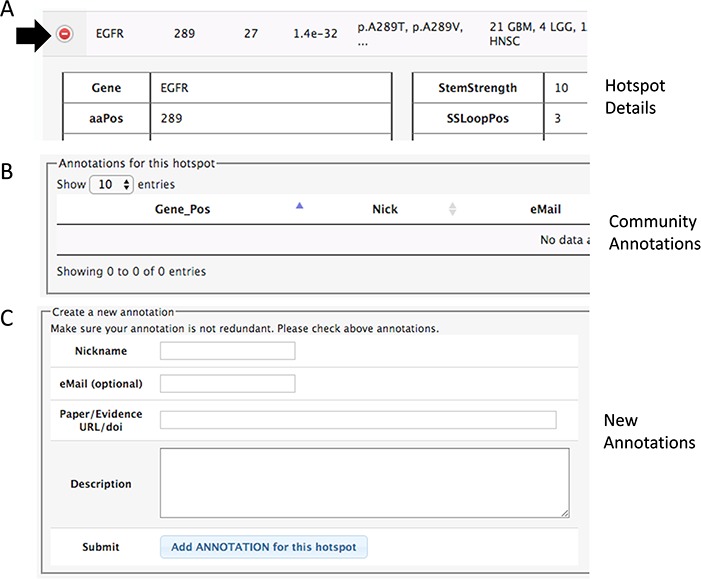
Section of the details to add community annotations. (**A**) Option to show details. (**B**) Current community annotations for that hotspot. (**C**) Form to add new user annotations.

It has been proposed that genes carrying passenger mutations follow a neutral selection model of evolution while genes carrying driver mutations follow positive selection (12). Cancer genes that carry an excess of driver mutations can be detected by high values of dN/dS, the ratio of non-synonymous mutations to synonymous mutations normalized by corresponding sites of non-synonymous sites or synonymous sites. Here, the cancer data were used for *de novo* estimations of dN/dS using the method proposed by Martincorena *et al.* (12) providing a powerful approximate of a gene-level selective pressure. As a validation, the mean values of dN/dS for missense, non-sense, splicing and indels were 1.06, 0.99, 0.99 and 0.98 respectively while the medians were 1.05, 0.84, 0.84 and 0.78. These values are very close to 1, which is the expectation for neutrality. Supplementary Figure 1 shows a comparison of the estimated values for genes under high positive selection. This figure confirms that the *de novo* estimations are consistent to those in Martincorena *et al.* (12).

## Database web interface

The information of the generated database can be viewed and downloaded from the website http://bioinformatica.mty.itesm.mx/HotSpotAnnotations. The web tool consists basically of two web pages (Figure 1A). The first shows the estimated hotspots (Figure 1B). Columns can be used to sort data. Search text can be used for filtering. All data in tables can be exported and downloaded to several formats. The information of a hotspot can be further detailed using the ‘(+)’ sign. The information includes the amino acid position and changes, number of mutations, transcript, p- and q-values of the beta-binomial model, summary of mutation types, summary of cancer types and the annotation of APOBEC3A hairpin target and dN/dS.

The second page is specific for a gene. This page can be accessed from the first page after selecting a hotspot. The gene-specific page shows six sections (Figure 2). The first section is a plot of amino acid positions in the x-axis and mutations in the y-axis (Figure 2A). The second section shows a similar plot highlighting positions instead of mutations (Figure 2B). The third section shows the estimated hotspots for the gene (Figure 2C). The fourth section shows a table of the fitting information of the model to estimate hotspots showing the observed mutations per site, the mutations explained by the beta-binomial model, those mutations explained by the fixed effect, and the probability of occurrence (Figure 2D). Within this section, a graphical representation of the model is also shown (Figure 2E). The fifth section shows the distribution of mutation per cancer type and consequences (Figure 2F). Finally, the sixth section shows all mutations for the gene (Figure 2G). This figure shows the results for EGFR as a representative example.

### Community hotspot annotation

In both pages within the HotSpots tab, the research community can annotate additional information associated with hotspots by opening the details using the ‘(+)’ option (Figure 3). The required information includes a URL or DOI referring to the evidence supporting the claim, a text description of the experiments, concepts or algorithms related to positive validation or negative confirmation as a hotspot, and optionally the user e-mail, id and nickname of the user.

## Discussion and conclusion

Hotspots mutations are commonly thought as driver mutations because it seems highly unlikely that the same residue is mutated in different patients. Nevertheless, it has been shown recently that hairpin formation in during DNA replication may be targeted by the APOBEC3A enzyme (18). This discovery provides direct evidence that passenger mutations in cancer may accumulate under certain circumstances to form passenger hotspots. Consequently, it is necessary to inform researchers of this evidence that may be useful when designing experiments regarding hotspots. To provide more information, *dN/dS*, a common and powerful measure from molecular evolution that may inform a possible neutral selection of the gene, was also included (12). Moreover, it was implemented the possibility to add manual annotations that document the experimental work regarding the associated hotspot providing further help to users. Altogether, the included information may aid users to encourage laboratory experiments or focus efforts in more promising hotspots.

Other hotspots have been proposed that are also passenger because of its correlation with higher mutation rates (4) or excessive gene sizes such as those in olfactory receptor genes, titin and mucins. Although this is highly plausible in the sense of background mutations rates, there is no mechanistic evidence that explains the appearance of specific hotspots in these highly mutated regions. To determine hotspots, a recent approach proposes a similar statistical model with covariates that show potential to avoid calling false hotspots (30). Nevertheless, besides it is pending to show experimental false positive and negative rates, this algorithm does not provide mechanistic evidence of the mutation bias such as in the APOBEC3A case. Thus, it is expected that novel experimental proposals will provide information on other passenger hotspot mechanisms. This topic will be tracked to further annotate the database.

To the time this work was performed, this is the first database dedicated to annotating hotspots and document researchers for possible passenger mechanistic artifacts. This may help to focus research efforts in hotspots having potential to drive oncogenic processes.

## Supplementary Material

Supp-Figure-1_baaa025

## Notes

**Database URL:** http://bioinformatica.mty.itesm.mx/HotSpotsAnnotations.
