# Supplementary figures and images for: HotSpotAnnotations—a database for hotspot mutations and annotations in cancer
Source: Database (Oxford). 2020 May 8;2020:baaa025. doi: 10.1093/database/baaa025 (PMC7211031; doi:10.1093/database/baaa025)

## Slide 1
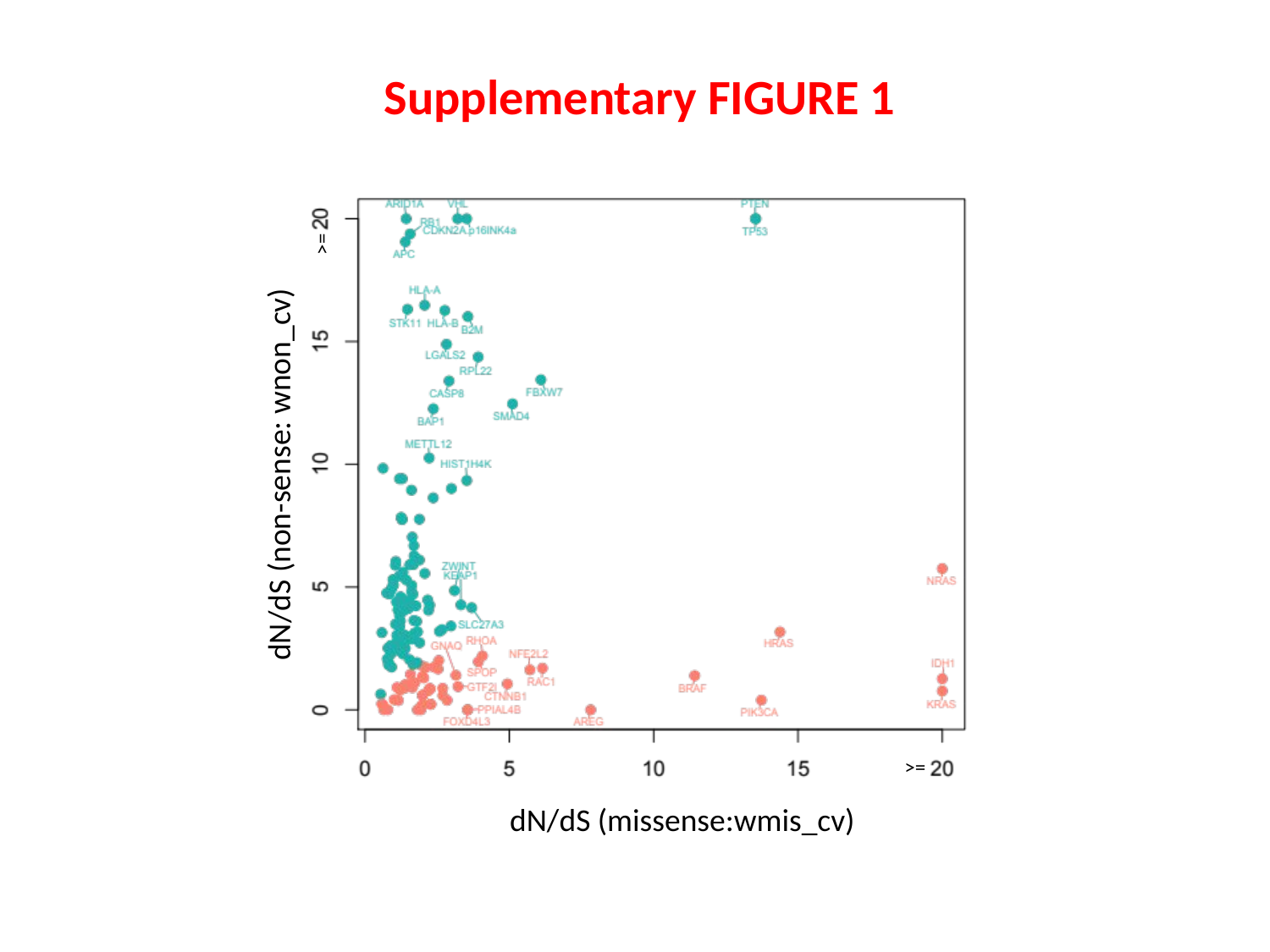

Supplementary FIGURE 1
>=
dN/dS (non-sense: wnon_cv)
>=
dN/dS (missense:wmis_cv)

Supplement: Supp-Figure-1_baaa025 [file supp-figure-1_baaa025.pptx]
